# The Effect of Phenolic-Rich Extracts of *Rubus fruticosus*, *R. ulmifolius* and *Morus nigra* on Oxidative Stress and Caco-2 Inhibition Growth

**DOI:** 10.3390/nu16091361

**Published:** 2024-04-30

**Authors:** Mariana S. Martins, Márcio Rodrigues, José David Flores-Félix, Cristina Garcia-Viguera, Diego A. Moreno, Gilberto Alves, Luís R. Silva, Ana C. Gonçalves

**Affiliations:** 1 CICS-UBI—Health Sciences Research Centre, University of Beira Interior, 6201-001 Covilhã, Portugal; mariana.sofia.morgado.martins@ubi.pt (M.S.M.); marciorodrigues@fcsaude.ubi.pt (M.R.); jdflores@usal.es (J.D.F.-F.); gilberto@fcsaude.ubi.pt (G.A.); 2 Research Unit for Inland Development, Polytechnic Institute of Guarda (UDI-IPG), 6300-654 Guarda, Portugal; 3 Microbiology and Genetics Department, University of Salamanca, 37007 Salamanca, Spain; 4 Laboratorio de Fitoquímica y Alimentos Saludables (LabFAS), Department Food Science and Technology, CSIC, CEBAS, Campus Universitario 25, Espinardo, 30100 Murcia, Spain; cgviguera@cebas.csic.es (C.G.-V.); dmoreno@cebas.csic.es (D.A.M.); 5 SPRINT—Sport Physical Activity and Health Research & Innovation Center, Instituto Politécnico da Guarda, 6300-559 Guarda, Portugal; 6 CERES, Department of Chemical Engineering, University of Coimbra, 3030-790 Coimbra, Portugal; 7 CIBIT—Coimbra Institute for Biomedical Imaging and Translational Research, University of Coimbra, 3000-548 Coimbra, Portugal

**Keywords:** blackberries, mulberries, phenolics, antioxidant potential, antiproliferative activities

## Abstract

Currently, a clear interest has been given to berries due to their richness in active metabolites, including anthocyanins and non-coloured phenolics. Therefore, the main aim of the present work is to investigate the phenolic profile, antioxidant abilities, and antiproliferative effects on normal human dermal fibroblasts (NHDF) and human colon carcinoma cell line (Caco-2) cells of phenolic-rich extracts from three red fruits highly appreciated by consumers: two species of blackberries (*Rubus fruticosus* and *Rubus ulmifolius*) and one species of mulberry (*Morus nigra*). A total of 19 different phenolics were identified and quantified by HPLC-DAD-ESI/MS^n^ and HPLC-DAD, respectively. Focusing on the biological potential of the phenolic-rich extracts, all of them revealed notable scavenging abilities. Concerning the antiproliferative properties, *R. fruticosus* presented a cytotoxic selectivity for Caco-2 cells compared to NHDF cells. To deeper explore the biological potential, combinations with positive controls (ascorbic acid and 5-fluorouracil) were also conducted. Finally, the obtained data are another piece of evidence that the combination of phenolic-rich extracts from natural plants with positive controls may reduce clinical therapy costs and the possible toxicity of chemical drugs.

## 1. Introduction

Red fruits, like blackberries (*Rubus* spp.) and mulberries (*Morus* spp.), are largely appreciated by consumers, not only due to their organoleptic properties but also due to their nutritional values [1,2,3]. Therefore, their daily intake is increasing worldwide, making it a hot topic of discussion among scientific and medical communities in order to fully explore their biological activities [4].

In fact, it is widely accepted that both berry fruits exert notable antioxidant, antimicrobial, and anti-inflammatory effects, as well as the ability to act as anti-ageing and antiproliferative agents, prevent cardiovascular pathologies, and boost the immune system [2,5,6]. In addition, they also showed antidiabetic properties; particularly, blackberries already showed the capability to increase fat oxidation and improve insulin sensitivity in overweight or obese males [5], while the hydro-alcoholic extract of *Morus nigra* reduced fasting blood glucose and haemoglobin A1c% in diabetic patients through competitive and allosteric interactions with the *α*-glucosidase enzyme [7]. In addition, they also display promising data regarding neurological protection: in fact, blackberry juice (8.7 mL/kg) already demonstrated capacity to diminish anxiety in Wistar rats [8], while *Morus nigra* juice (50 mg/kg) attenuated levodopa-induced dyskinesia in 1-methyl-4-phenyl-1,2,3,6-tetrahydropyridine-induced Parkinson’s disease in a mouse model [9]. Focusing on cancer, *M. nigra* can prolong the survival of rats with hepatocellular carcinoma at 400 mg/kg [6] and promote cell death by decreasing mutant p53 expression in HT-29 human colon cancer cells [10]. On the other hand, 15 mg/kg anthocyanins from *Rubus* blackberries showed potential to reduce hepatocellular carcinoma proliferation in HepG2 tumour-bearing mice [2].

As would be expected, their phenolic content plays a key role in their health-promoting qualities, particularly the presence of cyanidin 3-*O*-glucoside (Cy3Gluc), whose capabilities to control weight, reduce anti-inflammatory markers, offer protection against UVB-induced epidermal damage, and modulate hepatic gene expression, including promoting apoptosis in human hepatocellular carcinoma HepG2 cells, are already well-described [11,12]. In addition, these berries also present considerable amounts of quercetin, one of the most phenolic known, owing to their notable antioxidant, anti-inflammatory, antidiabetic, and antiproliferative effects [13].

Indeed, and also knowing that most synthetic drugs originate from several unwanted effects, it is not surprising the crescent incorporation of natural compounds and plant extracts in new pharmaceutical formulations [14,15]. Natural products are easier to obtain and more economical [16], and although they suffer rapid metabolism and present low bioavailability, many searches involving their (nano)-encapsulation have been conducted to improve it [14,15,17].

The present study aims to encourage the use of berry phenolic-rich extracts as coadjuvants to counteract oxidative stress levels and block cancer proliferation, as well as to analyse the economic savings, safety, and benefits that a combined therapy can offer. In this work, the phenolic profile of phenolic-rich extracts obtained by solid-phase extraction from two species of blackberries (*R. fruticosus* and *R. ulmifolius*) and one species of mulberry (*M. nigra*) collected in Covilhã (Portugal) was identified by HPLC-DAD-ESI/MS^n^ and quantified by HPLC-DAD. Furthermore, there was also evaluated, for the first time, the antioxidant abilities of phenolic-rich extracts from Portuguese blackberries and mulberries alone and combined with positive controls (ascorbic acid and 5-fluorouracil) against 1,1-diphenyl-2-picrylhydrazyl, nitric oxide, and superoxide radicals (DPPH^●^, ^●^NO and O_2_^●−^, respectively), as well as their potential to interfere with the growth of human colorectal adenocarcinoma Caco-2 cell lines. For comparative purposes, the effects of each extract on the viability of normal human dermal fibroblast NHDF cells were also tested.

## 2. Materials and Methods

### 2.1. Standards and Reagents

All chemicals used were of analytical grade. Cyanidin 3-*O*-glucoside (Cy3Gluc), cyanidin 3-*O*-rutinoside (Cy3Rut), pelargonidin 3-*O*-rutinoside (Pg3Rut) and peonidin 3-*O*-rutinoside (Pn3Rut) were from Extrasynthese (Genay, France). *N*-(1-Naphthyl)ethylenediamine dihydrochloride, sulfanilamide, 4-nitrophenyl-alpha-Dglucopyranoside, and sodium nitroprusside dihydrate (SNP) were obtained from Alfa Aesar (Karlsruhe, Germany). Trypsin-ethylenediaminetetraacetic acid (trypsin-EDTA) solution, 3-(4,5-dimethylthiazol-2-yl)-2,5-diphenyltetrazolium bromide (MTT), dimethyl sulfoxide (DMSO), and sodium nitroprusside dihydrate (SNP) were obtained from Alfa Aesar (Karlsruhe, Germany). Other phenolics and reagents were purchased from Sigma-Aldrich (St. Louis, MO, USA). Cell lines were from the American Type Culture Collection (ATCC, Manassas, VA, USA). Methanol and acetonitrile were from Fisher Chemical (Glenfield, Leicestershire, UK). Water was deionized using a Milli-Q water purification system (Millipore Ibérica, S.A.U., Madrid, Spain).

### 2.2. Sample Collection

**Figure 1:**
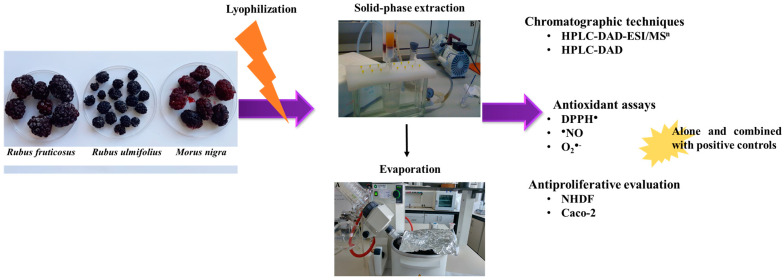
Summary figure regarding the extraction and analysis of *Rubus fruticosus* and *R. ulmifolius* blackberries and *Morus nigra* mulberry grown in Covilhã region, Portugal.

Approximately 1 kg of cultivated blackberry (*R. fruticosus*) and mulberry (*M. nigra*) were collected in July 2022, while blackberry from brambles (*R. ulmifolius*) was collected at the end of August 2022 in the Covilhã region, Portugal. All fruits were harvested at the commercial stage. The fruit collection was carried out manually, with gloves, placed immediately in plastic bags, and transported to Health Science Research Centre (CICS-UBI) facilities at low temperatures, to be rapidly frozen with liquid nitrogen and stored at −80 °C, to be further lyophilized (SCANVAC CoolSafetm, Frilabo, Portugal) and powdered (Figure 1).

### 2.3. Phenolic Compounds Extraction

In order to explore the antioxidant and antiproliferative potential of phenolics from blackberries and mulberries, their extraction was performed according to a method already described [18]. The yield of extraction was 22.44 ± 0.11% for *R. fruticosus*, 47.4 ± 0.25% for *R. ulmifolius*, and 28.15 ± 0.10% for *M. nigra*.

### 2.4. HPLC Analysis

#### 2.4.1. Phenolics Identification

Phenolic extracts from blackberries and mulberries were identified via HPLC-DAD-ESI/MS^n^, based on a previous method already described [19]. Phenolics were tentatively identified based on their elution order, retention times, and ultraviolet-visible and mass spectra features as compared to authentic standards analysed under the same conditions and data available in the literature [2,19,20,21,22,23].

#### 2.4.2. Phenolics Quantification

Phenolics quantification was conducted by HPLC-DAD using a Shimadzu LC-2010A HT Liquid Chromatography system (Shimadzu Corporation, Kyoto, Japan) using a Nucleosil^®^ 300 C18 column (250 × 4.6 mm; 5 µm particle size waters; MZ-Analysentechnik GmbH, Mainz, Germany), according to Gonçalves and co-workers [18]. Anthocyanins and non-coloured phenolics were identified by comparing their retention times and ultraviolet-visible spectra in the 200–600 nm range with the library of spectra previously compiled by the authors and by external standards at 520 nm for anthocyanins, 350 nm for flavonols, 320 nm for hydroxycinnamic acids, and 280 nm for tannins and hydroxybenzoic acids.

### 2.5. Biological Potential

#### 2.5.1. Antioxidant Potential

The antioxidant capacity of these berries to scavenge DPPH^●^, ^●^NO, and O_2_^●−^ was evaluated using the phenolic-rich extracts obtained by solid-phase extraction and assessed spectrophotometrically using a Microplate Spectrophotometer Reader (Bio-Rad Laboratories, Hercules, CA, USA), according to Gonçalves et al. [18]. In the three assays, ascorbic acid was used as a positive control, and each assay was performed using seven concentrations for each extract and conducted, at least, in triplicate. The obtained results were expressed as 25% inhibitory concentration (IC_25_) or half-maximal inhibitory concentration (IC_50_) values (µg/mL).

Similar conditions were also applied to study the potential effect of phenolic-rich fractions combined with ascorbic acid at different conditions (25:75, 75:25, and 50:50).

#### 2.5.2. Antiproliferative Potential

##### Cell Culture Conditions and Treatments

Normal human dermal fibroblasts (NHDF) and Caco-2 cells were cultured in 75 cm^2^ culture flasks and incubated at 37 °C in a humidified atmosphere of 5% CO_2_. NHDF cells were cultured in RPMI 1640 medium supplemented with 10% FBS, 2 mM L-glutamine, 10 mM HEPES, 1 mM sodium pyruvate, and 1% penicillin/streptomycin, while Caco-2 cells were cultured as a monolayer in DMEM supplemented with 20% FBS and 1% penicillin/streptomycin. In order to evaluate the antiproliferative effects, 200 µL of NHDF and Caco-2 cells were seeded at a density of 1.0 × 10^4^ cells and 2.5 × 10^4^ cells per mL, respectively [18,24], and after one day of incubation, both cell lines were treated with 200 µL of six different concentrations of *R. fruticosus*, *R. ulmifolius*, and *M. nigra* extracts (50 to 800 μg/mL) dissolved in the complete cell medium for another 24 h. After this time, the medium was completely removed, and the viability of the cells was assessed by MTT assay. Untreated cells were used as a control and 5-FU (0.65, 6.50, and 65 µg/mL) as positive control [25]. In addition, the most promising phenolic-rich extract was mixed with 5-FU to see possible synergic effects on Caco-2 cells. A total of six independent experiments per extract (or positive control), at least, were performed. For the several assays, NHDF cells were used between passages 14 to 19, and for Caco-2 cells, from 39 to 48.

##### 3-(4,5-Dimethylthiazol-2-yl)-2,5-diphenyltetrazolium Bromide (MTT) Assay

The metabolic activity of cells was evaluated via their capacity to reduce the yellow MTT (0.5 mg/mL in the appropriate serum-free medium) into a blue formazan product after 4 h of incubation at 37 °C. Therefore, after 24 h of cells’ exposure to each phenolic-rich extract, the medium of each well was removed and twice washed with 200 µL of PBS. Then, for 4 h, MTT was added. After this time, the MTT-containing serum-free medium was removed and the formazan crystals were dissolved using dimethyl sulfoxide [18]. The absorbance was measured at 570 nm using a microplate reader and a Bio-Rad Xmark spectrophotometer (Bio-Rad Laboratories, Hercules, CA, USA). The values of cell proliferation were expressed as percentages based on the relative absorbance measured in the treated wells versus the control wells.

### 2.6. Statistical Analysis

All data were recorded as the mean ± standard deviation of, at least, triplicate determinations. Mean values were compared using one-way analysis of variance (one-way ANOVA), and the means were classified by Tukey’s test at a 95% level of significance. Differences were considered significant at *p* < 0.05. To determine the contribution of the total phenolic compounds to the antioxidant activity shown by *R. fruticosus, R. ulmifolius,* and *M. nigra*, Pearson’s correlation coefficients were calculated. All analyses were performed using Graph Pad Prism Version 8.4.3 (GraphPad Software, Inc., San Diego, CA, USA).

## 3. Results and Discussion

### 3.1. Phenolic Profile

**Figure 2:**
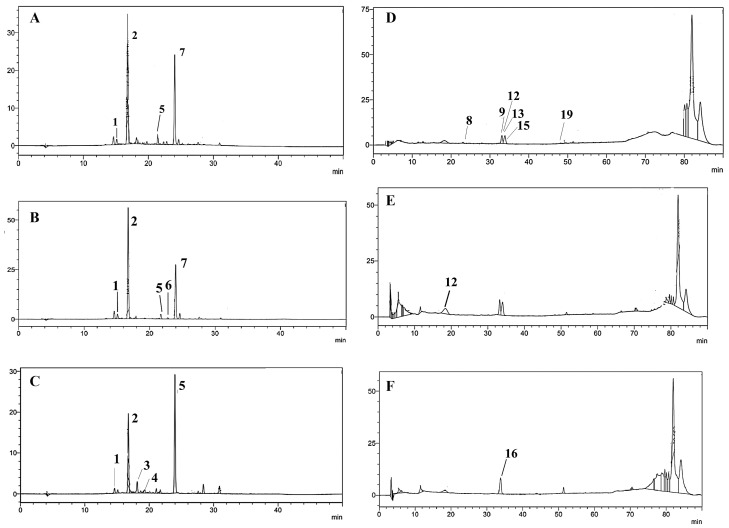
Anthocyanins found by HPLC-DAD at 520 nm in: (**A**), *R. fruticosus* phenolic-rich extract; (**B**), *R. ulmifolius* phenolic-rich extract; and (**C**), *M. nigra* phenolic-rich extract. Non-coloured phenolics obtained by HPLC-DAD at 350 nm found in: (**D**), *R. fruticosus* phenolic-rich extract; (**E**), *R. ulmifolius* phenolic-rich extract; and (**F**), *M. nigra* phenolic-rich extract. (1) cyanidin 3-*O*-glucoside (1), (2) cyanidin 3-*O*-glucoside (2), (3) cyanidin 3-*O*-rutinoside, (4), Pelargonidin 3-*O*-glucoside, (5) cyanidin arabinose/xyloside, (6) cyanidin-malonyl-glucoside, (7) cyanidin-dioxalyl-glucoside, (8) ellagitannin (pedunculagin I), (9) ellagitannin (Pedunculagin II), (12) ellagic acid pentoside (13) galloyl-hexahydroxydiphenoyl-glucoside, (15) quercetin 3-*O*-glucuronide, (16) quercetin 3-glucoside derivative and (19) quercetin 3-pentoside.

The phenolic profile of phenolic-rich extracts from Portuguese blackberries and mulberries was characterised, for the first time, by chromatographic techniques. A total of 19 different phenolic compounds were tentatively identified based on the interpretation of their fragmentation patterns obtained from mass spectra and by comparison with other published data [2,19,20,21,22,23] (Table S1). Particularly, there were seven anthocyanins, six flavonols, one hydroxycinnamic acid, three hydroxybenzoic acids, and two tannins found. The identified phenolics were quantified using HPLC-DAD (Table 1 and Figure 2A–F). The outcomes are consistent with previous research [2,3,16,20,21,22,23,26]; Cy-malonyl-glucoside, Cy-dioxalyl-glucoside, and galloyl-hexahydroxydiphenoyl-glucoside were also found in blackberry and mulberry fruits in a previous work (data not yet published). When comparing anthocyanin levels, a total of six anthocyanins were observed for *R. fruticosus* and *R. ulmifolius*, and five were found in *M. nigra* (Table 1 and Table S1), varying between 31.98% (*R. fruticosus*.) and 89.05% (*R. ulmifolius*) of the total level of phenolic compounds. Among the three species, (2) Cy3Gluc was the most abundant; *R. ulmifolius* had the highest concentration of this compound, accounting for around 62.53% of its total phenolics. In addition, Cy-malonyl-glucoside and Cy-dioxalyl-glucoside were only detected in *Rubus* spp. The presence of Cy 3-*O*-dioxalylglucoside, Cy 3,5-diglucoside, and Pg3Rut in *R. fruticosus* is in accordance with other studies, as is the presence of Cy3Gluc and Pn3Gluc in *R. fruticosus* and *M. nigra* [3,16].

Comparing with other red fruits, blueberries present other anthocyanins not detected in these berries, namely Dp 3-*O*-arabinoside (2147.51–4444.20 µg/g dw), Pt 3-*O*-galactoside (3609.50–19,654.66 µg/g dw), and malvidin 3-*O*-arabinoside (2249.05–3020.86 µg/g dw) [27], while sweet cherry phenolic extracts showed considerable amounts of Cy3Rut (3865.64 µg/g dw) and Pg3Rut (337.46 µg/g dw) [18].

The majority of anthocyanins can be found in red fruits, including grapes, strawberries, blueberries, and other red fruits and vegetables. Due to the large number of free hydroxyl groups surrounding the ring B, they are recognised as the main antioxidant molecules in the human diet [21]. In blackberries and mulberries, anthocyanins contribute about 90% of the antioxidant capacity [28], being also recognised due to their notable antibacterial, anti-inflammatory [29], and neuroprotective properties [30], as well as cellular signalling activity, cardiovascular [1], cancer prevention, and anti-diabetic activities [31], and being able to manage weight [32]. These effects are mainly attributable to anthocyanins’ ability to easily scavenge reactive species, chelate metals, and interact with proteins and active receptors on the peroxisome proliferator, modifying its activity and influencing substrate metabolism and inflammation [33,34]. All of these capabilities help to improve pathologic dangers such as cancer, diabetes, and cardiovascular diseases [4].

Relatively to non-coloured phenolics, *R. fruticosus* phenolic-rich extract had the highest concentration of non-coloured phenolic compounds (79,071.82 µg/g dw), followed by *M. nigra* (18,518.37 µg/g dw), and *R. ulmifolius* (6975.72 µg/g dw).

Focusing on the obtained data for *Rubus* species, it is important to highlight the presence of (12) ellagic acid pentoside and (13) galloyl-hexahydroxydiphenoylglucoside in both blackberries and of (15) quercetin (Q) 3-*O*-glucuronide (42,995.9 ± 539.94 µg/g dw) in *R. fruticosus*. On the other hand, the Q3Gluc derivative was the main non-coloured phenolic-rich fraction observed in *M. nigra*, representing 36.10% of total phenolic levels. Tannins, standing out in the presence of (8) ellagitannin (pedunculagin I) and (9) ellagitannin (pedunculagin II), were only detected in *R. fruticosus* phenolic-rich extract at amounts of 1151.40 and 9964.30 µg/g dw, respectively.

According to the literature, the presence of hydroxycinnamic acids, flavonols, and tannins in blackberries and mulberries increases their health benefits [35]. Although the only hydroxycinnamics identified in the current study were neochlorogenic acid and 5-*ρ*-coumaroyl quinic acid, other studies have already reported the additional presence of caffeic, ferulic, and hydroxybenzoic derivative acids [16,36,37].

In contrast with other small fruits, phenolic-rich extracts from sweet cherries present higher amounts of phenolic acids (with a total of non-coloured phenolics of 11,034.15 µg/g dw), representing about 69.8% of their total phenolic compounds [18], while Q aglycone was the main one detected in phenolic-rich blueberries (6962.43–7521.47 µg/g dw) [27].

The observed discrepancies between the results and the literature are to be expected, given they are mostly related to variations in genotypes, origin, soil, treatments, and processing [35,38].

### 3.2. Biological Potential

Numerous studies indicate that these perishable fruits directly contribute to their noteworthy health advantages. Previous studies on its antioxidant, anti-inflammatory, and brain-boosting properties found a clear correlation between these properties and high phenolic component concentrations, especially when anthocyanins were present [39]. Considering these facts, the current study examined the capacity of *R. fruticosus, R. ulmifolius*, and *M. nigra* phenolic-rich extracts to scavenge DPPH^●^, ^●^NO, and O_2_^●−^, as well as to interfere with the growth of NHDF cells and Caco-2 carcinoma cells.

#### 3.2.1. Antioxidant Activity

**Figure 3:**
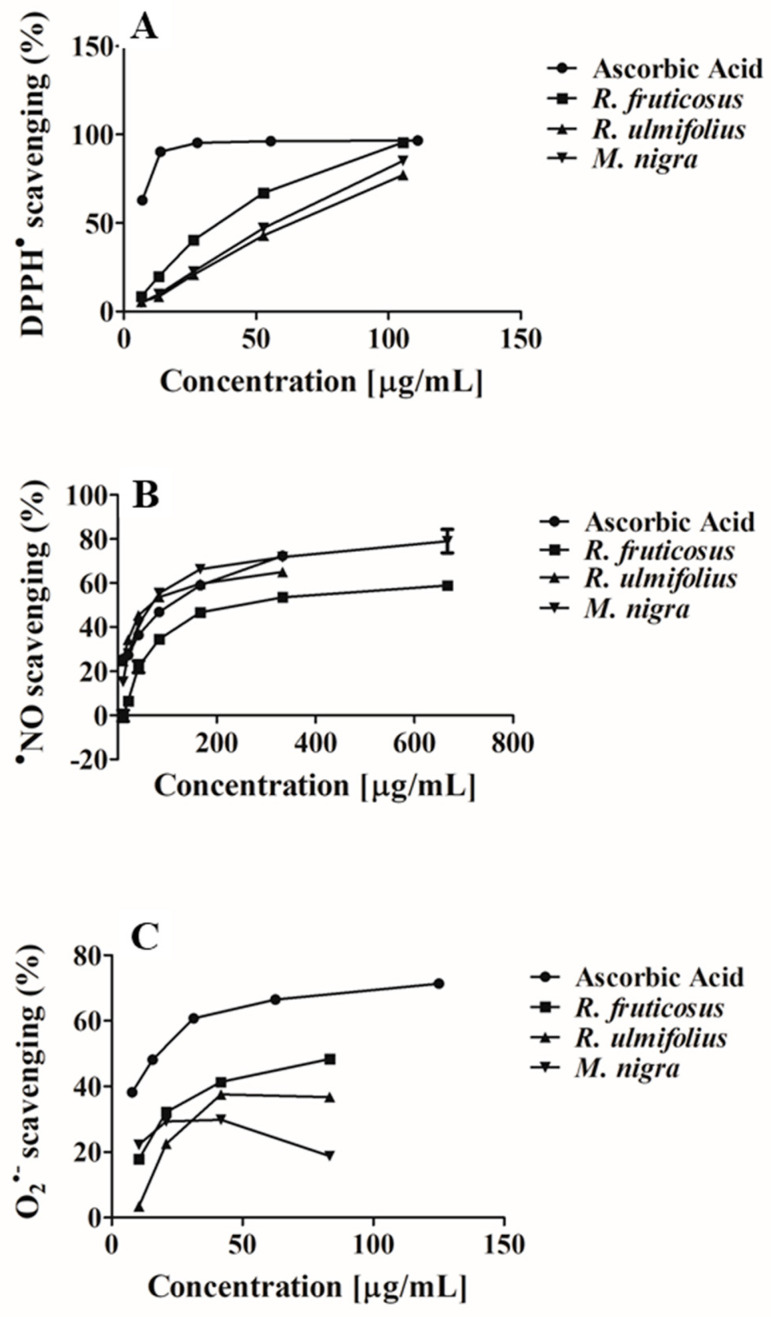
Antioxidant potential of *R. fruticosus*, *R. ulmifolius*, and *M. nigra* phenolic-rich extracts against (**A**) DPPH^●^, (**B**) ^●^NO and (**C**) O_2_^●−^.

Free radicals and reactive species play an indisputable role in human metabolism; however, their overproduction and accumulation lead to lipid, protein, and DNA damage, as well as necrosis and exacerbated inflammatory responses, which in turn promote the onset of several diseases, including cancer and cardiovascular and neurological pathologies [18]. Phenolics have previously shown great potential for lowering oxidative stress levels with little to no negative side effects, in contrast to synthetic antioxidants, which are regarded as dangerous and have unwanted side effects. As far as we know, phenolics are effective in controlling oxidative stress and restoring redox homeostasis by neutralising and/or reducing free radicals and the formation of reactive species, chelating trace elements involved in the formation of these pro-oxidant species, modulating related enzymes in cell signalling cascades, and stimulating the endogenous defence system [28]. As already mentioned, the antioxidant properties of the phenolic-rich extracts from blackberry and mulberry extracts were evaluated against DPPH^●^, ^●^NO, and O_2_^●−^ species. The obtained values are represented in Table 2 and Figure 3A–C.

DPPH is a synthetic radical; its test is frequently carried out because of its stability and simplicity. This makes it possible to broadly evaluate various extracts’ and individual components’ antioxidant activity. It is based on the transformation from violet to yellow that occurs when a tested substance or extract donates hydrogen to DPPH^●^, neutralising it [39]. All tested extracts exhibited dose-dependent effects against DPPH^●^ (Table 2 and Figure 3A). *R. fruticosus* was the extract most effective in reducing this radical, with an IC_50_ score of 34.29 ± 0.55 µg/mL dw, followed by *M. nigra* (IC_50_ value of 56.30 ± 0.96 µg/mL dw). The least active species was *R. ulmifolius* (IC_50_ = 62.55 ± 0.82 µg/mL dw). Nevertheless, all tested extracts displayed lower activity than the ascorbic acid-positive control (IC_50_ = 5.53 ± 0.40 µg/mL).

The capacity of *M. nigra* and *R. fruticosus* to reduce DPPH^●^ has already been reported [26,39,40]. Indeed, Huo and co-workers revealed that crude extracts of Chinese black *M. nigra* were the most effective (12.52 µmol Trolox equivalent (TE)/g dw), followed by the anthocyanin fraction (87.25 µmol TE/g dw) and the non-anthocyanin fraction (113.52 µmol TE/g dw) [39]. On the other hand, Turkish black *M. nigra* displayed IC_50_ values lower than those obtained in this study, ranging from 17.22 to 25.45 µg/mL [40]. Focusing on *R. fruticosus,* Polish blackberry *n*-hexane, carbon dioxide, and ethanol extracts showed IC_50_ scores of 2.47, 14.22, and 1223.84 µmol TE/g, respectively [41]. When compared to other red fruits, the investigated extracts were more efficient than blueberry extracts (IC_50_ values from 144.68 to 208.06 µg/mL) [27].

Nitric oxide (NO) is a chemical mediator produced by endothelial cells that is involved in several physiological effects to protect the organism against vascular, gastrointestinal, and nervous system vasodilation, as well as tumoural, microbial, and inflammatory processes [42]. However, excessive production has negative effects on proteins and mitochondria, activating pro-inflammatory transcription factors that promote inflammation and promoting the development of neurodegenerative and chronic diseases like cancer, diabetes, atherosclerosis, rheumatoid arthritis, and inflammatory bowel diseases [43]. Phenolic-rich extracts of *R. fruticosus*, *R. ulmifolius,* and *M. nigra* have also shown the ability to capture this radical in a dose-dependent manner (Table 2 and Figure 3B). Comparing the studied extracts, *R. ulmifolius* had the highest activity (IC_50_ = 59.49 ± 0.81 µg/mL dw), being nearly 1.7 times more potent than the ascorbic acid positive control (IC_50_ = 104.10 ± 0.96 µg/mL), followed by *M. nigra* (IC_50_ = 65.01 ± 0.63 µg/mL dw) and *R. fruticosus* (IC_50_ = 202.98 ± 2.12 µg/mL dw).

Relatively to other red fruits, the capacity to reduce ^●^NO was stronger than that of Sweetheart cherry phenolic-rich extract (IC_50_ = 358.64 ± 2.40 µg/mL dw) [27]. On the other hand, Saco sweet cherry extracts demonstrated superior activity (IC_50_ = 33.72 ± 0.89 µg/mL dw) [18]. In addition, *R. ulmifolius* exhibited similar activity compared to Legacy and Duke blueberry phenolic-rich extracts (50.34 and 69.53 µg/mL dw, respectively) [27].

The present study also assessed the ability of the phenolic-rich extracts from blackberries and mulberries to scavenge O_2_^●−^, since NO can react with O_2_^●−^, producing more toxic free radical species, such as hydrogen peroxide. This radical mainly results from purine metabolism and electron leakage from the respiratory chain [18]. As well as other radicals, O_2_^●−^ also plays a significant role in gene expression, signal transduction pathways, growth regulation, and immunological responses [18]. However, this one is also active at greater levels in a variety of pathophysiologic events, including inflammation, oxygen toxicity, and phagocyte-mediated activity. In the present study, the analysed extracts were also effective in scavenging this radical in a dose-dependent manner (Table 2 and Figure 3C). *M. nigra* phenolic-rich extract was the most active (IC_25_ = 14.26 ± 0.47 μg/mL) dw, followed by *R. fruticosus* (IC_25_ = 14.70 ± 0.58 μg/mL dw) and *R. ulmifolius* (IC_25_ = 23.59 ± 0.73 μg/mL dw). Even so, the three extracts showed lower activity than the ascorbic acid-positive control (IC_25_ = 3.19 ± 0.30 μg/mL). Only the IC_25_ was possible to determine.

Comparing with other berry phenolic-rich extracts, phenolic-rich extracts from Duke and Legacy blueberries were more effective, showing better IC_25_ values around 1.00 µg/mL [27].

In a general way, fruits’ antioxidant capacity is generally proportional to their phenolic content. Accordingly, it was feasible to identify positive correlations (r > 0.30; *p* > 0.05) between the DPPH^●^ values and the content of cyanidin derivatives quantified. However, despite the extracts’ potential to trap NO^●^, negative correlations were discovered between the concentrations of Cy3Gluc (1) and (2) (r < −0.20) and Cy arabinose/xyloside (r < −0.50) and their capacity to quench this radical. In addition, strong positive correlations (r > 0.90; *p* > 0.05) were discovered between Cy derivatives and the assay to capture O_2_^●−^. In general, the presence of anthocyanins and non-coloured compounds does not directly influence the capture of free radicals. In addition, in most cases, the highest content of phenolics leads to increased antioxidant activity. Indeed, the chemical structure of phenolics, namely those composed of catechol, pyrogallol, and methoxy groups, confers on them an easy capacity to neutralise free radicals. Even so, the presence of other non-determined bioactive compounds that are able to interact in additive or synergistic ways with phenolics cannot be ignored.

##### Antioxidant Mixtures

Despite potential synergistic effects, phenolic-rich extracts and ascorbic acid were combined to further the evidence gained (Table 3). One of the most notable antioxidants and anti-inflammatory substances is ascorbic acid, also known as vitamin C, which is found in higher concentrations in citrus fruits, particularly oranges. This molecule is considered essential for the health of the human body owing to its role in a number of physiological processes, such as blood vessel strengthening and sealing, controlling leukocyte microbial absorption, lowering cholesterol levels, and speeding up the healing of wounds [44]. This molecule also controls the synthesis of collagen, slows down the ageing process of the skin, and lowers blood pressure. Due to all these notable properties, this one is largely incorporated in supplements and pharmaceutical formulations to promote a healthy status. In fact, clinical evidence has been reported that topical treatment with ascorbic acid alleviates the symptoms of skin ageing and increases the production of collagen [45].

When comparing the results, it is evident that ascorbic acid and phenolic-rich extracts typically interact synergistically to increase the antioxidant activities of the mixture. Focusing on *R. fruticosus* as an example, its phenolic-rich extract alone had activity against DPPH^●^ of IC_50_ = 34.29 ± 0.55 µg/mL, but when combined with ascorbic acid (50:50), it was almost four times more effective (IC_50_ = 9.42 ± 0.20 µg/mL). Additionally, an increment against ^●^NO was also observed combining *R. ulmifolius* phenolic-rich extracts and an ascorbic acid-positive control. In particular, its combination with ascorbic acid (50:50) results in lower values of IC_50_ when compared to the control ascorbic acid alone (IC_50_ values of 28.27 ± 0.40 and 104.10 ± 0.96 µg/mL, respectively). On the other hand, all the mixtures had an antagonistic effect against O_2_^●−^, with IC_25_ values higher than those of the ascorbic acid control (IC_25_ = 3.19 ± 0.30 µg/mL). Taking into account the unintentional side effects of synthetic pharmaceutical products, the interest in natural products and nutraceuticals has increased worldwide to be incorporated into pharmaceuticals or even to replace certain drugs with natural alternatives.

#### 3.2.2. Antiproliferative Activity

##### Normal Human Dermal Fibroblasts (NHDF)

**Figure 4:**
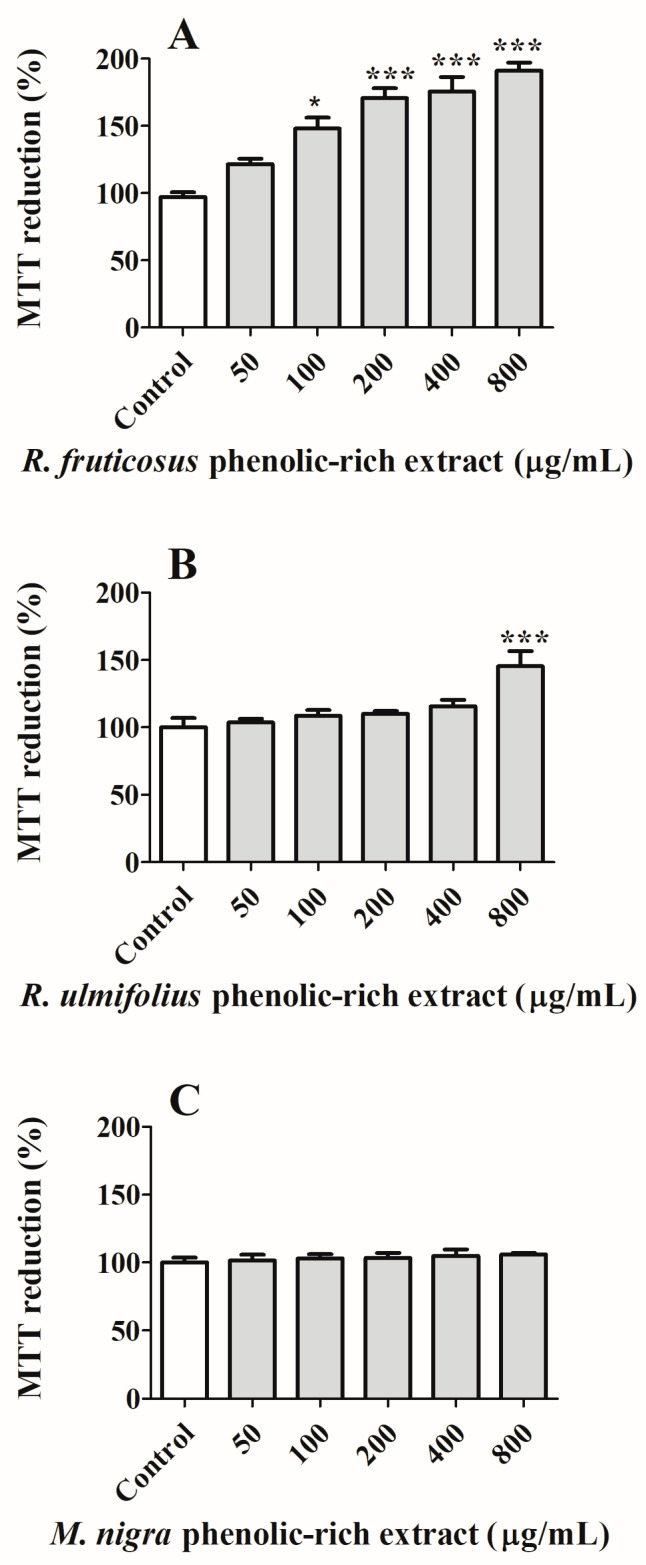
Effect of (**A**) *R. fruticosus* and (**B**) *R. ulmifolius* blackberries, and (**C**) *M. nigra* mulberry phenolic-rich extracts on NHDF viability after 24 h of exposure, assessed by MTT reduction. Values show mean ± standard deviation of six independent assays, at least, performed in triplicate (* *p* < 0.05, *** *p* < 0.0001 compared to the respective controls).

A preliminary cytotoxicity study using NHDF cells was carried out in order to select non-toxic concentrations for a non-tumourous cell line (Figure 4). Similar assays are routinely performed [46,47]. Analysing the obtained data, it is possible to observe that the tested concentrations (50–800 µg/mL) do not exhibit any form of toxicity for normal cells. The increment in NHDF cell proliferation can be related to the antioxidant properties shown by natural extracts, as well as their richness in growth factors, nutrients, and bioactive compounds and their ability to promote cell cycle progression by regulating the expression and activity of cell cycle-related proteins and modulating the extracellular matrix [46,47].

##### Human Colon-Rectal Adenocarcinoma (Caco-2) Cells

**Figure 5:**
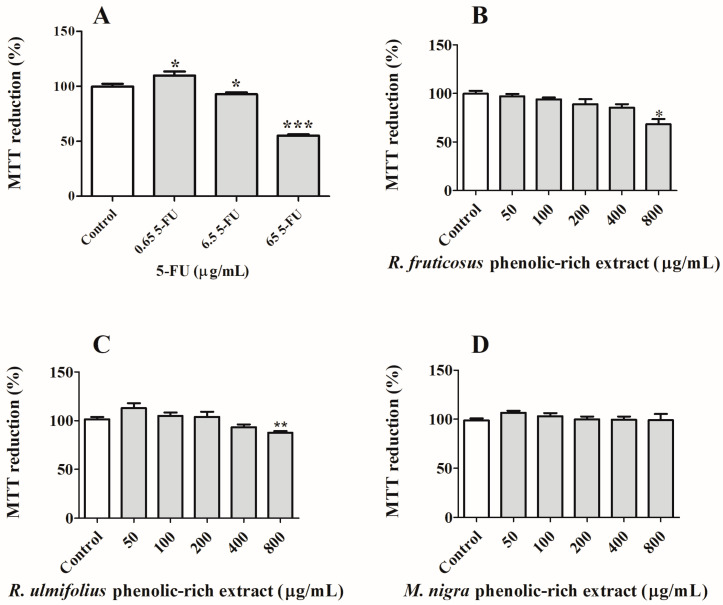
Effects of (**A**) anti-tumoural drug 5-FU, (**B**) *R. fruticosus* and (**C**) *R. ulmifolius* blackberries, and (**D**) *M. nigra* mulberry phenolic-rich extracts on Caco-2 cells viability after 24 h of exposure, assessed by MTT reduction. Values show mean ± standard deviation of six independent assays, at least, performed in triplicate (* *p* < 0.05, ** *p* < 0.01 and *** *p* < 0.0001 compared to the respective controls).

Taking into consideration the concentrations of extracts that showed no cytotoxic effects on NHDF cells, their effects against Caco-2 viability were then studied (Figure 5). This cell line was chosen because it is a well-known model of the intestinal epithelium and because, after differentiation, it forms monolayers that mimic a number of intestinal epithelial cell properties [48]. In addition, it is largely used in the in vitro model of colorectal cancer, and it is also important to note that, after berry intake, their compounds contact the intestinal epithelium [18]. After 24 h of exposure, it is feasible to compare the effects of phenolic-rich extracts to see how they affect Caco-2 cells’ proliferation, and only *R. fruticosus* revealed the most notable activity at the highest dose examined (800 µg/mL). However, the antiproliferative activity was less effective compared to that demonstrated by the anti-tumoural drug 5-FU at 65.0 µg/mL. Additionally, the phenolic-rich extract of *R. ulmifolius* showed a slight cytotoxic activity, while *M. nigra* did not exhibit any kind of anti-growth ability for these cancer cells at the assessed time of exposure. However, it is expected that the antiproliferative effects of the extracts would rise with an increase in the duration of their exposure to cancer cells.

Considering the obtained data from this study, it was possible to conclude that, out of the three species, only *R. fruticosus* can interfere with the proliferation of Caco-2 cells, most likely because of its high anthocyanin concentration. Indeed, the presence of phenolics, particularly anthocyanins, is associated with this ability. In fact, the coloured extract of sweet cherry extracts was revealed to possess more notable activity to prevent Caco-2 cell proliferation than their non-coloured extract, showing an IC_50_ value of 667.84 µg/mL, promoting necrosis in the highest concentrations tested (>200 µg/mL) [18]. Even so, particular attention has been given to ellagitannins, since red raspberry (*Rubus idaeus*) ellagitannins also displayed the capacity to affect the nuclear morphology and induce the apoptosis of Caco-2 cells (IC_50_ score of 124 µg/mL) [49].

Of course, it is important to note that these results are preliminary, and in humans, the efficacy of natural matrices and compounds is highly dependent on several factors (e.g., gender, age, lifestyle, genetics, existence or note of pathologies, cooking processes, chemical structures, and so on) that can highly influence the bioavailability and bioaccessibility [50]. In addition, the gut microbiota also assumes a notable role, given its ability to enhance the absorption of compounds, particularly anthocyanins [50,51,52]. To improve their efficacy, new strategies involving (nano)-encapsulation have been conducted [14,15,17].

##### Combination of *R. fruticosus* with 5-FU Anti-Cancer Drug

**Figure 6:**
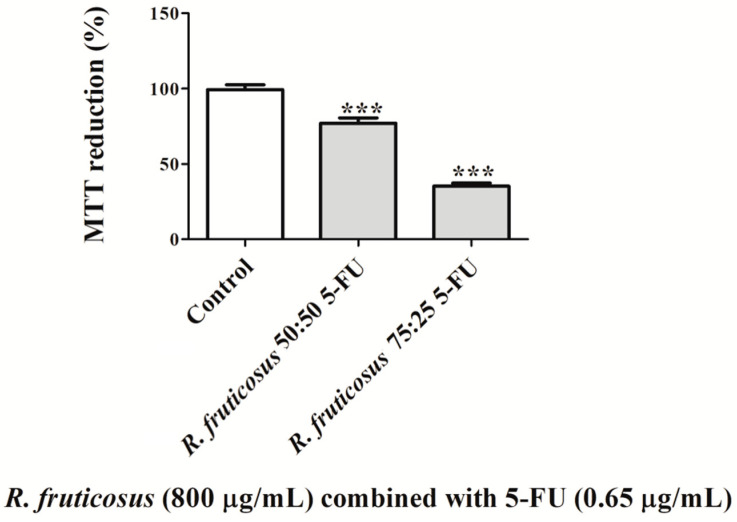
Effects of combined 50:50 and 75:25 of *R. fruticosus* (800 µg/mL) and 5-FU (0.65 µg/mL) anti-cancer drug on Caco-2 cells for 24 h. After that time, cells’ viability was assessed by MTT reduction. Values show mean ± standard deviation of six independent assays, at least, performed in triplicate (*** *p* < 0.0001 compared to the respective control).

5-Fluorouracil (5-FU) is a powerful anti-cancer drug frequently used to treat several malignancies, including colon and breast cancers. This drug is a heterocyclic aromatic organic compound with a structure similar to that of the pyrimidine molecules of DNA and RNA, being an analogue of uracil with a fluorine atom at the C-5 position in place of hydrogen [53]. This chemotherapeutic drug has been used because it can inhibit the growth of cancer cells, but long-term use can have unwanted side effects [54]. Knowing the current interest and the possible incorporation of plant-based products in anti-cancer drugs [55], in the present study, the highest concentration of the most promising phenolic-rich extract (*R. fruticosus*, 800 µg/mL) was mixed with the lowest concentration of the anticancer drug 5-FU (0.65 µg/mL). These concentrations were chosen because 800 µg/mL demonstrated the most notable antiproliferative effects, and 0.65 µg/mL of 5-FU was the minimum concentration studied in order to see the antitumour potential of the extract when the drug is in low concentration compared to the nutraceutical. Therefore, mixtures of *R. fruticosus* and 5-FU (75:25 and 50:50) were made and tested (Figure 6). The mixture of extract and 5-FU (25:75) was not evaluated because the major goal is to decrease the concentration of the 5-FU synthetic drug to diminish its unwanted side effects and also its price.

The findings collected indicated that there is a synergistic impact (combination index < 1) between *R. fruticosus* and 5-FU, which enhances the anticarcinogenic potential of each one. A similar study assessed the anti-cancer potential of thiosulfinate-enriched *Allium sativum* extract in combination with 5-FU chemotherapy against the growth of Caco-2 cells. It was shown that this combination is quite beneficial, boosting the potential of each one [25]. Given that synthetic medications used for therapy have negative side effects and consequences for the human body, this investigation is interesting since natural products, including *R. fruticosus* berry, are easily available in the environment and do not damage humans.

## 4. Conclusions

The use and interest of natural products for therapeutic purposes have been increasing lately, especially those derived from plants and rich in antioxidant molecules, namely phenolic compounds. This occurs because they are inexpensive and are believed to have fewer side effects and lower toxicity than synthetic pharmaceutical drugs. In the present study, 19 phenolic compounds were found, highlighting the presence of Cy3Gluc. *R. ulmifolius* was the one with the highest concentration of anthocyanins, while *R. fruticosus* was the richest one in non-coloured phenolics. Regarding the biological potential, the phenolic-rich extract from *R. fruticosus* was the most active against DPPH^●^, while *R. ulmifolius* was the most active against ^●^NO. On the other hand, phenolic-rich extracts from both *M. nigra* and *R. fruticosus* exhibited similar and intriguing activity in the O_2_^●−^. Concerning the antiproliferative properties, *R. fruticosus* was the most promising, inhibiting Caco-2 cells’ growth. Additionally, the mixture of *R. fruticosus* extract with 5-FU at ratios of 50:50 and 75:25 demonstrated a considerable increase in this activity, which may open the way to combining this drug already used against tumours with blackberries to enhance the antiproliferative effects. Based on the available data, it can be concluded that blackberries and mulberries have strong antioxidant properties, which may be beneficial for treating oxidative-related diseases such as cardiovascular, inflammatory, and tumour pathologies. This discovery is another argument in favour of including blackberries and mulberries in pharmaceutical and nutraceutical formulations, although further study, especially animal studies and then clinical trials, is needed to fully examine the biological potential and safe dose. Furthermore, although bioavailability and bioaccessibility are believed to be greater than expected, mainly due to the action of gut microbiota, it is also imperative to explore the bioavailability and bioaccessibility of these berries in the human body.

## Figures and Tables

**Table 1:** Quantification of anthocyanins and non-coloured phenolic compounds (µg/g of dried fruit) identified in *Rubus fruticosus* and *R. ulmifolius* blackberries and *Morus nigra* mulberry phenolic-rich extracts grown in Covilhã region, Portugal.

Peaks	Phenolic Compounds	*R. fruticosus* L.	*R. ulmifolius* Schott	*Morus nigra*
Anthocyanins				
1	Cy3Gluc (1)	946.79 ± 27.48	2224.40 ± 81.70 ^a^	185.31 ± 11.87 ^b^
2	Cy3Gluc (2)	24,053.14 ± 34.88	39,847.30 ± 73.40 ^a^	13,252.31 ± 60.59 ^ab^
3	Cy3Rut	nq	nd	735.13 ± 113.50
4	Pg-Gluc	nd	nq	119.41 ± 32.40
5	Cy arabinose/xyloside	264.65 ± 10.43	329.95 ± 16.23	14,554.56 ± 262.51 ^ab^
6	Cy-malonyl-gluc	nq	13,766.39 ± 161.81	nd
7	Cy-dioxalyl-gluc	11,919.49 ± 117.57	579.96 ± 24.63	nd
	**Ʃ**	**37,184.06**	**56,748.00**	**28,846.72**
Non-coloured phenolic compounds				
8	Ellagitannin (Pedunculagin I)	1151.40 ± 41.95	nd	nd
9	Ellagitannin (Pedunculagin II)	9964.30 ± 107.04	nd	nd
10	NEO	nd	nq	nd
11	5-*ρ*-CQA	nd	nd	nq
12	EA pentoside	9348.74 ± 109.27	6975.72 ± 547.83 ^a^	nd
13	Galloyl-hexahydroxydiphenoyl-gluc	11,382.10 ± 56.26	nq	nd
14	Q3R	nq	nd	nq
15	Q3-*O*-glucuronide	42,995.9 ± 539.94	nd	nd
16	Q3G derivative	nq	nq	18,518.37 ± 370.71
17	Q acetyl-gluc	nd	nd	nq
18	K3R	nd	nd	nq
19	Q3-pentoside	4229.38 ± 123.21	nd	nd
	**Ʃ**	**79,071.82**	**6975.72**	**18,518.37**

Cy: cyanidin; Gluc: glucoside; Rut: rutinoside; Pg: Pelargonidin; NEO: Neochlorogenic acid; CQA: Coumaroyl quinic acid; EA: Ellagic acid; Q: Quercetin; K: Kaempferol. Values are expressed as mean ± standard deviation of three assays. ∑, sum of the determined anthocyanins; nd; not detectable; nq, not quantified. Significant differences between different extracts were obtained by Tukey’s test (*p* < 0.05) and indicated by: ^a^: *Rubus fruticosus* L. phenolic-rich extract; ^b^ *Rubus fruticosus* L. phenolic-rich extract.

**Table 2:** Antioxidant capacity of *Rubus fruticosus* and *Rubus ulmifolius* Schott blackberries and *Morus nigra* blueberry phenolic-rich extracts grown in Covilhã region, Portugal. Values were expressed as **25% inhibitory concentration (IC_25_)** or half-maximal inhibitory concentration (IC_50_) values (μg/mL dry weight).

Assay	*Rubus fruticosus* L.	*Rubus ulmifolius* Schott	*Morus nigra* L.
DPPH^●^ (IC_50_)	34.29 ± 0.55	62.55 ± 0.82 ^a^	56.30 ± 0.96 ^a^
^●^NO (IC_50_)	202.98 ± 2.12	59.49 ± 0.81 ^a^	65.01 ± 0.63 ^a^
**O_2_^●−^** **(IC_25_)**	**14.70 ± 0.58**	**23.59 ± 0.73 ^a^**	**14.26 ± 0.47 ^b^**

Values are expressed as mean ± standard deviation of three assays concerning the antioxidant capacity against 1,1-diphenyl-2-picrylhydrazyl, nitric oxide and superoxide radicals (DPPH^●^, ^●^NO and O_2_^●−^, respectively). Significant differences between phenolic-rich extracts according to Tukey’s test (*p* < 0.05) are indicated by: ^a^ vs. *R. fruticosus*; ^b^ vs. *R. ulmifolius*.

**Table 3:** Antioxidant capacity of *R. fruticosus* and *R. ulmifolius* blackberries and *M. nigra* mulberry phenolic-rich extracts grown in Covilhã region, Portugal combined with ascorbic acid positive control. Values were expressed as **IC_25_** or IC_50_ values (µg/mL).

Assay	Extract			
	Ascorbic Acid	*Rubus fruticosus* L.	*Rubus ulmifolius* Schott	*Morus nigra* L.
DPPH^●^	25:75	10.89 ± 0.14	4.18 ± 0.20 ^a^	3.14 ± 0.18 ^ab^
	50:50	9.42 ± 0.20	6.65 ± 0.21 ^a^	3.32 ± 0.12 ^ab^
	75:25	6.77 ± 0.15	4.04 ± 0.28 ^a^	6.68 ± 0.19 ^b^
^●^NO	25:75	295.77 ± 0.87	80.29 ± 0.19 ^a^	184.36 ± 1.41 ^ab^
	50:50	227.28 ± 1.65	28.27 ± 0.40 ^a^	176.25 ± 0.85 ^b^
	75:25	19.66 ± 0.28	31.70 ± 0.71 ^a^	16.97 ± 0.88 ^b^
O_2_^●−^	**25:75**	**8.22 ± 0.41**	**7.73 ± 0.33**	**24.10 ± 0.63 ^ab^**
	**50:50**	**12.87 ± 0.23**	**8.19 ± 0.41**	**37.28 ± 1.01 ^ab^**
	**75:25**	**8.44 ± 0.20**	**13.11 ± 0.50**	**22.47 ± 0.98 ^ab^**

Values are expressed as mean ± standard deviation of three assays concerning the antioxidant capacity against 1,1-diphenyl-2-picrylhydrazyl, nitric oxide and superoxide radicals (DPPH^●^, ^●^NO and O_2_^●−^, respectively). ^a^ Significant result (*p* < 0.05) is indicated as vs. *Rubus fruticosus* L. ^b^ *p* < 0.05 is indicated as vs. *Rubus ulmifolius* Schott.

## Data Availability

Data presented in this study are available within the article.

## Supplementary Materials

The following supporting information can be downloaded at: https://www.mdpi.com/article/10.3390/nu16091361/s1, Table S1: Retention time (Rt), wavelengths of maximum absorption in the ultraviolet-visible region (λmax), mass spectral data and identification of anthocyanins and non-coloured phenolic compounds found in *R. fruticosus* and *R. ulmifolius* blackberries and *M. nigra* mulberry phenolic-rich extracts grown in Covilhã region, Portugal.
