# Supplementary material for: The Effect of Phenolic-Rich Extracts of Rubus fruticosus, R. ulmifolius and Morus nigra on Oxidative Stress and Caco-2 Inhibition Growth
Source: Nutrients. 2024 Apr 30;16(9):1361. doi: 10.3390/nu16091361 (PMC11085810; doi:10.3390/nu16091361)
Supplement: Supplementary file 1 [file nutrients-16-01361-s001.zip › nutrients-2988655-SI.pdf]

nutrients

MDPI

Supplementary Materials

The Effect of Phenolic-Rich Extracts of *Rubus fruticosus*, *R. ulmifolius* and *Morus nigra* on Oxidative Stress and Caco-2 Inhibition Growth

Mariana S. Martins [1], Márcio Rodrigues [1,2], José David Flores-Félix [1,3], Cristina Garcia-Viguera [4], Diego A. Moreno [4], Gilberto Alves [1], Luís R. Silva [1,5,6,*] and Ana C. Gonçalves [1,7,*]

Table S1. Retention time (Rt), wavelengths of maximum absorption in the ultraviolet-visible region (λmax), mass spectral data and identification of anthocyanins and non-coloured phenolic compounds found in *R. fruticosus* and *R. ulmifolius* blackberries and *M. nigra* mulberry phenolic-rich extracts grown in Covilhã region, Portugal.

**Peak**	**Phenolic compounds**	**HPLC-DAD-ESI-MS^n characteristics**			
		**Rt (min)**	**λ max**	**Molecular ion [M+H] (m/z)**	**Fragments MS^n (m/z)**
		Anthocyanins			
1	Cyanidin 3-*O*-glucoside (1)	7.5	518	449	287
2	Cyanidin 3-*O*-glucoside (2)	9.4	518	449	287
3	Cyanidin 3-*O*-rutinoside	10.2	518	595	449,287
4	Pelargonidin glucoside	11.5	-	433	287
5	Cyanidin arabinose/xyloside	13.2	518	419	287
6	Cyanidin-malonyl-glucoside	14.5	518	535	287
7	Cyanidin-dioxalyl-glucoside	15.8	516	593	287
		Non-coloured compounds			
8	Ellagitannin (Pedunculagin I)	4.1	376	783	481,301
9	Elagitannin (Pedunculagin II)	5.2	374	783	481,301
10	3-Caffeoyl-quinic acid (Neochlorogenic acid)	7.3	329	353	191,179
11	5-*ρ*-Coumaroyl quinic acid	11.2	320	337	191
12	Ellagic acid pentoside	14.9	364	433	301
13	Galloyl-Hexahydroxydiphenoyl-glucoside	16.8	360	633	301,275
14	Quercetin 3-*O*-rutinoside	17.3	364	609	301
**15**	**Quercetin 3-*O*-glucuronide**	**18.5**	**354**	**477**	**301**
16	Quercetin 3-*O*-glucoside derivative	20.7	354	603, 463	301
17	Quercetin acetyl-glucoside	20.8	354	505	301
18	Kaempferol 3-rutinoside	20.9	346	593	285
19	Quercetin 3-pentoside	21.3	356	433	301
